# Examining the role of industry lobbying on Canadian front-of-pack labelling regulations

**DOI:** 10.17269/s41997-024-00950-1

**Published:** 2024-10-11

**Authors:** Jennifer J. Lee, Emily R. Ziraldo, Hayun Jeong, Mary R. L’Abbé

**Affiliations:** 1 Department of Nutritional Sciences, Temerty Faculty of Medicine, University of Toronto, Toronto, ON Canada; 2 School of Nutrition, Faculty of Community Services, Toronto Metropolitan University, Toronto, ON Canada

**Keywords:** Front-of-pack labelling, Nutrition labelling, Regulations, Lobbying, Public health policy, Étiquetage de face, étiquetage nutritionnel, réglementation, pressions, politique de santé publique

## Abstract

Health Canada recently issued a Marketing Authorization to expand the eligibility of the dairy-related exemption for Canadian front-of-pack labelling (FOPL) regulations. The 2024 Marketing Authorization exempts dairy-related products that are a ‘source of calcium,’ rather than only ‘high in’ calcium as previously regulated, from displaying a ‘High in’ front-of-pack nutrition symbol, regardless of their saturated fat and sodium levels. The Marketing Authorization, heavily influenced by the food industry, lacks strong scientific evidence to support its adoption. Although there is a high prevalence of inadequate calcium intakes among Canadians, the Marketing Authorization will exempt more dairy-related products that are significant contributors of saturated fat and sodium for Canadians. While providing very little calcium, many dairy-related products, particularly cheese products, are ‘high in’ saturated fat and/or sodium. Expanding the exemption criteria will allow dairy-related products with little health benefits to be reflected as ‘healthy’ (i.e., not display a ‘High in’ nutrition symbol), blunting the potential impact that FOPL regulations could have on improving the diets of Canadians. We strongly urge Health Canada to reconsider the expansion of the exemption and encourage others to conduct policy-relevant research and participate in the policy decision-making process to promote evidence-informed public health policies for the health of Canadians.

## Résumé

Santé Canada a récemment publié une autorisation de mise en marché qui élargit l’admissibilité des produits laitiers à l’exemption au règlement canadien sur l’étiquetage sur le devant de l’emballage. L’autorisation de 2024 exempte les produits dérivés du lait qui sont « source de calcium », et non simplement « élevés en » calcium comme dans la réglementation antérieure, de l’exigence d’afficher le symbole nutritionnel « élevé en » sur le devant de l’emballage, et ce, peu importe leur teneur en gras saturés et en sodium. Très influencée par l’industrie alimentaire, cette autorisation de mise en marché ne repose pas sur des bases scientifiques solides. Bien que les apports insuffisants en calcium soient très prévalents dans la population canadienne, l’autorisation de mise en marché exemptera un nombre accru de produits dérivés du lait qui sont pourtant des sources importantes de gras saturés et de sodium dans l’alimentation canadienne. De nombreux produits dérivés du lait contenant très peu de calcium, en particulier des produits de fromage, sont en effet « élevés en » gras saturés ou en sodium. L’élargissement des critères d’exemption permettra à des produits dérivés du lait comportant très peu d’avantages pour la santé d’être perçus comme étant « sains » (en n’affichant pas le symbole nutritionnel « élevé en »), atténuant ainsi l’effet possible du règlement sur le régime des Canadiennes et des Canadiens. Pour le bien de la santé de la population, nous exhortons Santé Canada à changer d’avis sur l’élargissement de l’exemption et nous encourageons d’autres parties à mener des études et à participer au processus décisionnel sur la question de fond afin de promouvoir des politiques de santé publique éclairées par la science.

Poor diet is a leading modifiable risk factor for non-communicable diseases in Canada and worldwide (World Health Organization, 2018). In 2016, Health Canada launched the *Healthy Eating Strategy* to “*make the healthier choice the easier choice,*” which included the announcement of front-of-pack labelling (FOPL) regulations and a suite of additional policy initiatives targeting the food environment (Health Canada, 2016). FOPL has been shown to improve population diets by improving consumers’ purchasing behaviours (Taillie et al., 2021) and driving manufacturer-driven reformulation (Reyes et al., 2020). Canada’s FOPL regulations were first drafted in *Canada Gazette (CG) I* in 2018, and officially finalized and published in *CGII* in July 2022 for implementation by January 2026. In June 2024, Health Canada issued a Marketing Authorization to expand the exemption criteria for dairy-related products from FOPL regulations (Health Canada, 2024).

According to FOPL regulations, pre-packaged food and beverage products (hereinafter “products”) that meet and/or exceed thresholds for nutrients-of-concern (saturated fat, sugars, and sodium) are required to display a ‘High in’ nutrition symbol (Government of Canada, 2022). Thresholds for Canadian FOPL regulations are set based on the percent daily value (%DV) per reference amount (i.e., regulated serving size used in Canadian food labelling) for each nutrient-of-concern (i.e., 10%DV for products with a reference amount of ≤ 30 g or mL, 15%DV for products with a reference amount of > 30 g or mL; and 30%DV for main dishes with a reference amount of ≥ 200 g or ≥ 170 g if designed for 1 to < 4-year-old children). There are three exemption criteria, where products meeting the criteria would not display a ‘High in’ nutrition symbol, regardless of their nutrient levels: (i) health-; (ii) technical-; and (iii) practical-related exemption criteria (Government of Canada, 2022).

One of the most notable differences between *CGI* (i.e., draft) and *CGII* (i.e., final regulations) has been the expansion of the exemption criteria, where dairy-related products (i.e., cheese, kefir, yogurt, and buttermilk) that are ‘good’ or ‘excellent’ sources of calcium (i.e., ≥ 10%DV per reference amount for products with a reference amount of ≤ 30 g or mL; or ≥ 15%DV for products with a reference amount of > 30 g or mL) became eligible for exemption under the “health-related” exemption criteria (Government of Canada, 2022), due to inadequate calcium intakes among Canadians (Ahmed et al., 2021a). The 2024 Marketing Authorization further expands the exemption criteria for dairy-related products to include ‘sources’ of calcium (i.e., ≥ 5%DV per reference amount) (Health Canada, 2024). The dairy-related exemptions are scheduled to be examined 10 years following the implementation of FOPL regulations (i.e., 2036) (Government of Canada, 2022).

## Impact of lobbying on Canadian FOPL regulations

**Fig. 1:**
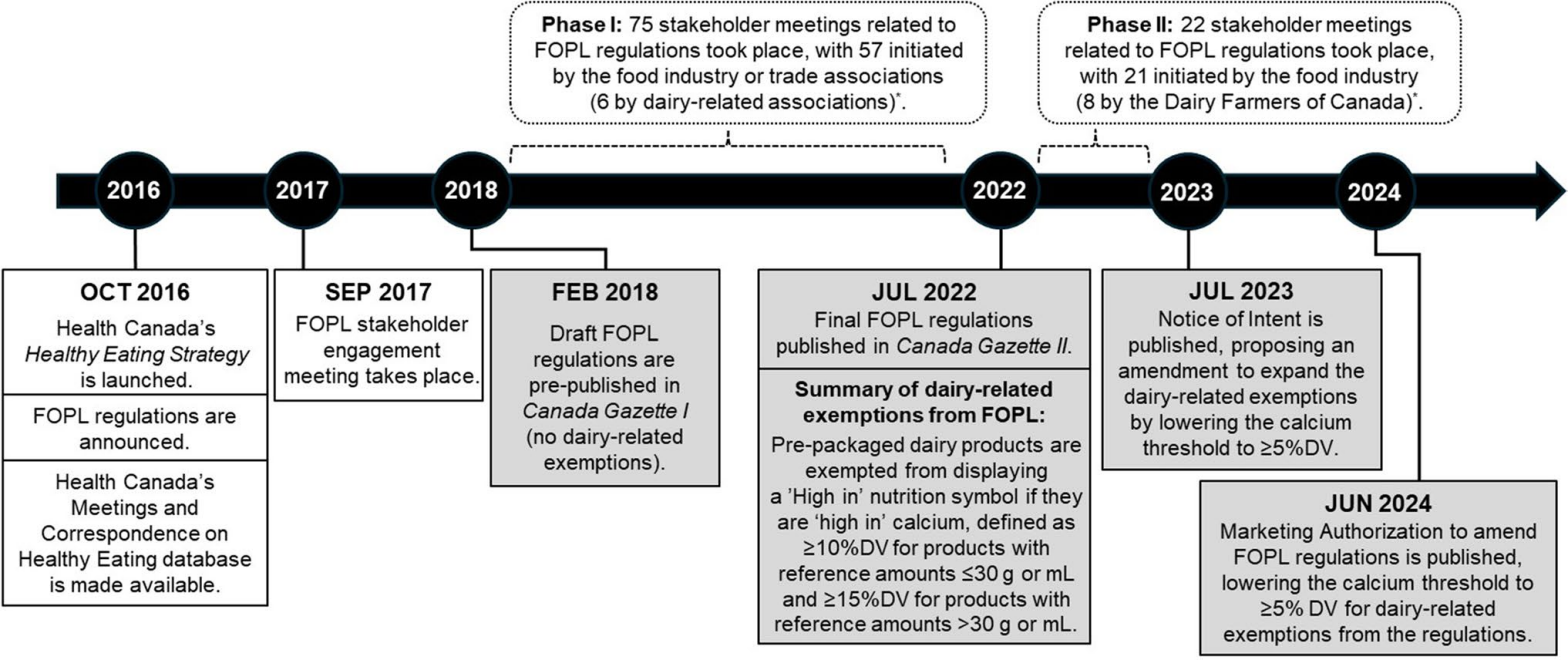
Overview of the events and activities related to Canadian front-of-pack labelling (FOPL) regulations. Canadian FOPL regulations require pre-packaged food and beverage products meeting and/or exceeding thresholds for nutrients-of-concern (saturated fat, sugars, and sodium) to display a ‘High in’ nutrition symbol on the front of the product package. Canadian FOPL regulations were first announced in 2016 as part of the *Healthy Eating Strategy*, issued in draft form in *Canada Gazette I* in 2018, and finalized in *Canada Gazette II* in 2022 for implementation by 2026. ^*^Health Canada’s Meetings and Correspondence on Healthy Eating Database was used to examine stakeholder-initiated meetings and correspondences. Abbreviations: FOPL, Front-of-pack labelling; %DV, Percent Daily Value

Although the decision-making process for regulatory changes has typically taken place behind closed doors, the launch of Health Canada’s Regulatory Transparency and Openness Framework has helped reveal the role of stakeholders in the regulatory development process. Using the data from Health Canada’s Meetings and Correspondence on Healthy Eating Database (Government of Canada, 2023), we reviewed each of the stakeholder-initiated meetings and correspondences (hereinafter “meetings”) related to FOPL regulations. Figure 1 shows an overview of regulatory events and activities related to FOPL regulations. Approximately 40% (n = 75/187) of the meetings that took place between February 2018 (*CGI* release) and June 2022 (*CGII* publication) were related to FOPL regulations with the majority initiated by the food industry or trade associations (n = 57/75), including six meetings initiated by the dairy-related associations. Half of the stakeholder-initiated meetings between June 2022 (*CGII* publication) and July 2023 (Notice of Intent issued to amend FOPL regulations (Health Canada, 2023)) were related to FOPL regulations (n = 22/44) and nearly all were initiated by the food industry (n = 21/22) with the Dairy Farmers of Canada as the most prevalent stakeholder (36%; n = 8/22). A review of these meeting notes between the Dairy Farmers of Canada and Health Canada revealed that concerns regarding FOPL regulations and cheese products were repeatedly raised prior to the publication of a Notice of Intent to expand the exemption criteria for dairy-related products (Government of Canada, 2023).

## Potential impact of lobbying on policy outcomes

Despite the high prevalence of calcium inadequacy among Canadians, with over 40% of adult males and over 60% of adult females having calcium intakes below the Estimated Average Requirement (Ahmed et al., 2021a), the expansion of the exemption criteria for dairy-related products will do little to address the inadequate calcium intakes among Canadians and is not supported by current evidence.

The top contributor of calcium for Canadians is milk, not cheese or yogurt, while cheese is a significant contributor of saturated fat and sodium. Data from the latest nationally representative dietary survey (Canadian Community Health Survey-Nutrition 2015) indicated that among the Milk and Alternatives food group (according to the food groups of 2007 Canada’s Food Guide), the top source of calcium was milk, contributing over 30% of the calcium intakes of Canadians (Auclair et al., 2019), and the top source of both saturated fat and sodium was cheese (Auclair et al., 2019). Cheese was the third highest source of saturated fat across all food categories for Canadians, contributing 6% of daily saturated fat consumption, and the 17th source of sodium, contributing 2% of daily sodium consumption (Kirkpatrick et al., 2019). However, the contribution of saturated fat and sodium intakes from cheese to overall intakes was likely higher if cheese consumed as ingredients in mixed products (e.g., pizza, sandwiches) was considered, as such analyses have previously identified cheese as the 4th highest source of sodium across all food categories for Canadians (Fischer et al., 2009). Therefore, expanding the exemption criteria would result in exempting dairy-related products that are minimal sources of calcium, but significant sources of saturated fat and/or sodium for Canadians, particularly cheese.

The newly introduced expansion of the exemption would lead to more dairy-related products with high levels of saturated fat and/or sodium not displaying a ‘High in’ nutrition symbol, which has the potential to seriously blunt the impact of FOPL regulations and confuse consumers. Using the University of Toronto’s Food Label Information and Price (FLIP) 2020, a nationally-representative, branded-food composition database (Ahmed et al., 2021b), 1172 products in the cheese, yogurt, and kefir food categories were examined according to *CGI, CGII,* and the 2024 Marketing Authorization. Table 1 shows the proportion of cheese, yogurt, and kefir that would be exempted or would need to display a ‘High in’ nutrition symbol. Under the 2024 Marketing Authorization, 84% (n = 984) of products would be exempted from displaying a ‘High in’ nutrition symbol—compared to previous FOPL regulations (i.e., *CGII*, n = 658)*,* this translates to a 43% decrease in ‘high in’ saturated fat (n = 171, down from 299) and a 41% decrease in ‘high in’ sodium products (n = 60, down from 102) not displaying the appropriate ‘High in’ nutrition symbol. This is particularly concerning as the exemptions from dairy-related products introduced in *CGII* have already resulted in the exemption of 658 products (56% of products in these categories) from displaying a ‘High in’ nutrition symbol for saturated fat and/or sodium. Table 2 shows the nutrient levels of products that would be exempted according to *CGII* and newly exempted according to the 2024 Marketing Authorization. Under both exemption criteria scenarios, exempted cheeses are ‘high in’ saturated fat, with an average of 4.5 g/serving (22%DV) and 3.4 g/serving (17%DV) according to *CGII* and the 2024 Marketing Authorization, respectively. However, cheeses that would be newly exempted according to the 2024 Marketing Authorization are not ‘high in’ calcium, with an average of 115.2 mg/serving (9%DV). Therefore, the 2024 Marketing Authorization expanding the exemption criteria for dairy-related products, particularly for cheese products, will allow many more products that are high in saturated fat and/or sodium with no additional health benefits (i.e., not ‘high’ in calcium) to be exempted.

**Table 1:** Summary of number and prevalence of dairy-related products categorized by thresholds of front-of-pack labelling regulations according to *Canada Gazette I, Canada Gazette II* and the 2024 Marketing Authorization

		*Canada Gazette I* Criteria (i.e., No exemptions)			*Canada Gazette II* Criteria (i.e., ‘good’ or ‘excellent’ source of calcium)			2024 Marketing Authorization Criteria (i.e., ‘source’ of calcium)		
TRA Category	n	Exempted^*^	Not exempted		Exempted^*^	Not exempted		Exempted^*^	Not exempted	
			‘High in’ saturated fat	‘High in’ sodium		‘High in’ saturated fat	‘High in’ sodium		‘High in’ saturated fat	‘High in’ sodium
**Cheese**	**736**	**0**	**661 (90%)**	**345 (47%)**	**452 (61%)**	**241 (33%)**	**102 (14%)**	**574 (78%)**	**153 (21%)**	**60 (8%)**
D.1—Cheese, all other	634	0	594 (94%)	316 (50%)	401 (63%)	220 (35%)	99 (16%)	477 (75%)	150 (24%)	58 (9%)
D.1.1—Cheese fondue preparation	2	0	2 (100%)	2 (100%)	0	2 (100%)	2 (100%)	0	2 (100%)	2 (100%)
D.2—Cottage cheese	30	0	6 (20%)	(0%)	3 (10%)	1 (3%)	0	29 (97%)	0	0
D.3—Cheese used as an ingredient	21	0	13 (62%)	(0%)	5 (24%)	13 (62%)	0	20 (95%)	0	0
D.4—Hard cheese	37	0	36 (97%)	26 (70%)	34 (92%)	3 (8%)	1 (3%)	37 (100%)	0	0
D.5—Quark, fresh cheese and fresh dairy desserts	12	0	10 (83%)	1 (8%)	9 (75%)	2 (17%)	0	11 (92%)	1 (8%)	0
**Yogurt and kefir**	**436**	**0**	**150 (34%)**	**0**	**206 (47%)**	**58 (13%)**	**0**	**410 (94%)**	**18 (4%)**	**0**
D.12—Fermented dairy drinks	54	0	13 (24%)	0	28 (52%)	3 (6%)	0	47 (87%)	3 (6%)	0
D.12.1—Drinkable yogurts	6	0	1 (17%)	0	0	1 (17%)	0	6 (100%)	0	0
D.15—Yogurt	376	0	136 (36%)	0	178 (47%)	54 (14%)	0	357 (95%)	15 (4%)	0
**TOTAL**	**1172**	**0**	**811 (69%)**	**345 (29%)**	**658 (56%)**	**299 (26%)**	**102 (9%)**	**984 (84%)**	**171 (15%)**	**60 (5%)**

n = 1172. Using the University of Toronto’s Food Label Information and Price 2020 database, food and beverage products in select Health Canada’s Table of Reference Amounts for Food (TRA) categories (Health Canada, 2022) were assessed according to front-of-pack labelling regulations in *Canada Gazette I* (draft regulations), *Canada Gazette II* (final regulations), and the 2024 Marketing Authorization (extending dairy-related exemptions). According to Canadian front-of-pack labelling regulations published in *Canada Gazette II,* dairy-related products (i.e., cheese, yogurt, kefir, and buttermilk) would be exempted from displaying a ‘High in’ nutrition symbol for saturated fat and sodium regardless of their nutrient levels for being ‘good’ or ‘excellent’ source of calcium, defined as having calcium levels ≥ 15%DV per reference amount for products with the reference amount of > 30 g or mL or ≥ 10%DV per reference amount for products with the reference amount of ≤ 30 g or mL (Government of Canada, 2022). The 2024 Marketing Authorization expands the exemption criteria to include dairy-related products that are a ‘source’ of calcium, defined as calcium levels ≥ 5%DV per reference amount (Health Canada, 2024). Buttermilk products (n = 3) were excluded from the analyses as they would not display a ‘High in’ nutrition symbol according to all three scenarios. ^*^Refers to products that would be exempted from saturated fat and sodium only, not sugars. Abbreviations: TRA, Table of Reference Amounts for Food; %DV, Percent Daily Value

**Table 2:** Nutrient content of dairy-related products that would be exempted from front-of-pack labelling regulations according to *Canada Gazette II* and newly exempted according to the 2024 Marketing Authorization

TRA Category	*Canada Gazette II* Criteria (i.e., ‘good’ or ‘excellent’ source of calcium)							2024 Marketing Authorization Criteria (i.e., ‘source’ of calcium)						
	n	Saturated fat		Sodium		Calcium		n	Saturated fat		Sodium		Calcium	
		g	%DV	mg	%DV	mg	%DV		g	%DV	mg	%DV	mg	%DV
**Cheese**	**452**	**4.5 ± 2.8**	**22%**	**171.4 ± 93.8**	**7%**	**233.4 ± 79.6**	**18%**	**122**	**3.4 ± 1.9**	**17%**	**231.1 ± 116.1**	**10%**	**115.2 ± 29.0**	**9%**
D.1—Cheese, all other	401	5.4 ± 1.7	27%	209 ± 78	9%	217 ± 60	17%	76	4.0 ± 1.6	20%	246 ± 122	11%	101 ± 14	8%
D.2—Cottage cheese	3	1.3 ± 0.1	7%	46 ± 9	2%	211 ± 8	16%	26	1.2 ± 0.6	6%	280 ± 50	12%	159 ± 18	12%
D.3—Cheese used as an ingredient	5	16.5 ± 11.8	83%	107 ± 130	5%	215 ± 9	19%	15	3.7 ± 0.9	18%	88 ± 45	4%	118 ± 28	9%
D.4—Hard cheese	34	4.5 ± 1.5	22%	287 ± 103	12%	253 ± 66	37%	3	2.7 ± 0.3	13%	240 ± 44	10%	103 ± 13	8%
D.5—Quark, fresh cheese and fresh dairy desserts	9	9.6 ± 5.6	48%	143 ± 157	6%	486 ± 370	19%	2	5.6 ± 7.6	28%	100 ± 91	4%	99 ± 16	8%
**Yogurt and kefir**	**206**	**2.4 ± 2.3**	**12%**	**84.4 ± 19.4**	**4**%	**252.9 ± 58.0**	**20**%	**204**	**2.1 ± 2.2**	**10**%	**76.2 ± 19.8**	**3%**	**175.4 ± 21.2**	**13%**
D.12—Fermented dairy drinks	28	1.9 ± 0.9	9%	101 ± 14	4%	257 ± 44	19%	19	1.5 ± 0.8	7%	74 ± 14	3%	169 ± 15	13%
D.12.1—Drinkable yogurts	0							6	1.9 ± 1.1	10%	62 ± 14	3%	149 ± 24	12%
D.15—Yogurt	178	2.5 ± 2.4	13%	82 ± 19	4%	252 ± 60	18%	179	2.2 ± 2.3	11%	77 ± 20	3%	177 ± 21	14%

Values are means ± SD. Using the University of Toronto’s Food Label Information and Price 2020 database, food and beverage products in select Health Canada’s Table of Reference Amounts for Food (TRA) categories (Health Canada, 2022) were assessed according to front-of-pack labelling regulations published in *Canada Gazette II* (final regulations) and the 2024 Marketing Authorization (extending dairy-related exemptions). According to Canadian front-of-pack labelling regulations published in *Canada Gazette II,* dairy-related products would be exempted from displaying a ‘High in’ nutrition symbol for saturated fat and sodium regardless of their nutrient levels for being ‘good’ or ‘excellent’ source of calcium, defined as having calcium levels ≥ 15%DV per reference amount for products with the reference amount of > 30 g or mL or ≥ 10%DV per reference amount for products with the reference amount of ≤ 30 g or mL (Government of Canada, 2022). The 2024 Marketing Authorization expands the exemption criteria to include dairy-related products that are a ‘source’ of calcium, defined as calcium levels ≥ 5%DV per reference amount (Health Canada, 2024). According to Canadian nutrition labelling regulations, “5% or less is **a little,** 15% or more is **a lot**” (Government of Canada, 2016). Abbreviations: TRA, Table of Reference Amounts for Food; %DV, Percent Daily Value

Further expanding the dairy-related exemption, as outlined in the 2024 Marketing Authorization, undermines the healthy eating guidelines of 2019 Canada’s Food Guide, sodium reduction policies, and the integrity of FOPL regulations. Canada’s Dietary Guidelines explicitly state in Guideline 1: “*Protein foods include legumes, nuts, seeds, tofu, fortified soy beverage, fish, shellfish, eggs, poultry, lean red meat including wild game, lower fat milk, lower fat yogurts, lower fat kefir, and cheeses lower in fat and sodium*” (Health Canada, 2019). However, the expansion of the exemption will not identify dairy-related products “lower” in fat and/or sodium. Further, despite Health Canada’s commitment to reduce sodium intakes of Canadians to an average intake of 2300 mg/day (Health Canada, 2016), progress towards sodium reduction has been minimal (Health Canada, 2018). In 2012, Health Canada published voluntary sodium reduction targets to reduce sodium levels in processed foods by 2016; however, their evaluation report showed that almost half of the food categories did not make any progress to reduce sodium (Health Canada, 2018). Although FOPL regulations would have complemented the updated 2020–2025 voluntary sodium reduction targets (Health Canada, 2020) and positively influenced food reformulation, the recent exemption criteria expansion may undermine their efforts and dissuade manufacturers from reformulating products to reduce sodium levels.

## Conclusion

A strong commitment to evidence-based decision-making and active stakeholder engagement by policy decision makers are essential to ensure that evidence-informed public health policies are implemented. Health Canada has taken six years to publish FOPL regulations despite evidence for its ability to improve population’s diets. However, the 2024 Marketing Authorization, amending FOPL regulations, appears to have been heavily influenced by lobbying efforts that lack scientific evidence of benefit and have the potential to weaken the effectiveness of FOPL regulations. We strongly urge Health Canada to reconsider the expanded dairy-related exemption, driven by the dairy industry lobbying, and commit to evidence-informed public health policies that can improve the health of Canadians. We also urge health professionals, research community members, and others to conduct policy-relevant research and participate in the policy decision-making process to promote strong, evidence-informed public health policies for the health of Canadians.

## Data Availability

Data described in the manuscript and analytic code will be made available upon request, pending application and approval.

## Code availability

Analytic code will be made available upon request, pending application and approval.

## References

[CR1] Ahmed, M., Ng, A., & L’Abbe, M. R. (2021a). Nutrient intakes of Canadian adults: Results from the Canadian Community Health Survey (CCHS)–2015 public use microdata file. *American Journal of Clinical Nutrition,**114*(3), 1131–1140. 10.1093/ajcn/nqab143 34020449 10.1093/ajcn/nqab143 PMC8408873

[CR2] Ahmed, M., Schermel, A., Lee, J. J., Weippert, M., Franco-Arellano, B., & L’Abbé, M. (2021b). Development of the food label information program: A comprehensive Canadian branded food composition database. *Frontiers in Nutrition,**8*, 825050. 10.3389/fnut.2021.825050 35187026 10.3389/fnut.2021.825050 PMC8852278

[CR3] Auclair, O., Han, Y., & Burgos, S. A. (2019). Consumption of milk and alternatives and their contribution to nutrient intakes among Canadian adults: Evidence from the 2015 Canadian community health survey—nutrition. *Nutrients,**11*(8), 1948. 10.3390/nu11081948 31430962 10.3390/nu11081948 PMC6724033

[CR4] Fischer, P. W. F., Vigneault, M., Huang, R., Arvaniti, K., & Roach, P. (2009). Sodium food sources in the Canadian diet. *Applied Physiology, Nutrition and Metabolism,**34*(5), 884–892. 10.1139/h09-077 10.1139/H09-077 19935850

[CR5] Government of Canada. (2016). Regulations Amending the Food and Drug Regulations (Nutrition Labelling, Other Labelling Provisions and Food Colours). https://gazette.gc.ca/rp-pr/p2/2016/2016-12-14/html/sor-dors305-eng.html. Accessed 4 Nov 2022.

[CR6] Government of Canada. (2022). *Regulations amending the food and drug regulations* (Nutrition Symbols, Other Labelling Provisions, Vitamin D and Hydrogenated Fats or Oils): SOR/2022–168. Government of Canada. https://canadagazette.gc.ca/rp-pr/p2/2022/2022-07-20/html/sor-dors168-eng.html. Accessed 3 Sep 2023.

[CR7] Government of Canada. (2023). Meetings and correspondence on healthy eating. https://www.canada.ca/en/health-canada/services/food-nutrition/healthy-eating-strategy/meetings-correspondence.html. Accessed 13 Dec 2023.

[CR8] Health Canada. (2016). *Healthy eating strategy*. https://www.canada.ca/content/dam/canada/health-canada/migration/publications/eating-nutrition/healthy-eating-strategy-canada-strategie-saine-alimentation/alt/pub-eng.pdf. Accessed 7 Mar 2020.

[CR9] Health Canada. (2018). Sodium reduction in processed foods in Canada: An evaluation of progress toward voluntary targets from 2012 to 2016. Health Canada. https://www.canada.ca/content/dam/hc-sc/documents/services/food-nutrition/legislation-guidelines/guidance-documents/guidance-food-industry-reducing-sodium-processed-foods-progress-report-2017/pub1-eng.pdf. Accessed 15 Feb 2023.

[CR10] Health Canada. (2019). *Canada’s dietary guidelines for health professionals and policy makers*. https://food-guide.canada.ca/en/guidelines/. Accessed 7 Mar 2020.

[CR11] Health Canada. (2020). Voluntary sodium reduction targets for processed foods 2020–2025. https://www.canada.ca/en/health-canada/services/food-nutrition/healthy-eating/sodium/sodium-reduced-targets-2020-2025.html. Accessed 15 Dec 2022.

[CR12] Health Canada. (2022). *Table of reference amounts for food*. https://www.canada.ca/en/health-canada/services/technical-documents-labelling-requirements/table-reference-amounts-food/nutrition-labelling.html. Accessed 24 Nov 2022.

[CR13] Health Canada. (2023). *Notice of intent regarding the Minister of Health’s intention to publish marketing authorizations to permit vitamin D fortification of yogurt and kefir and expand the eligibility for the dairy-related exemption from the front-of-package nutrition labelling requirement*. https://www.canada.ca/en/health-canada/services/food-nutrition/public-involvement-partnerships/notice-intent-marketing-authorizations-permit-vitamin-d-fortification-yogurt-kefir-expand-eligibility-dairy-related-exemption-front-of-package-nutrition-labelling-requirement.html. Accessed 29 Jul 2023.

[CR14] Health Canada. (2024). Marketing authorization to permit a lower calcium threshold for exemptions from the requirement for prepackaged products to carry a nutrition symbol in the case of cheese, yogurt, kefir and buttermilk: SOR/2024–89. https://canadagazette.gc.ca/rp-pr/p2/2024/2024-06-05/html/sor-dors89-eng.html. Accessed 16 May 2024.

[CR15] Kirkpatrick, S. I., Raffoul, A., Lee, K. M., & Jones, A. C. (2019). Top dietary sources of energy, sodium, sugars, and saturated fats among Canadians: Insights from the 2015 Canadian Community Health Survey. *Applied Physiology, Nutrition and Metabolism,**44*(6), 650–658. 10.1139/apnm-2018-0532 10.1139/apnm-2018-0532 30951373

[CR16] Reyes, M., Taillie, L. S., Popkin, B., Kanter, R., Vandevijvere, S., & Corvalán, C. (2020). Changes in the amount of nutrient of packaged foods and beverages after the initial implementation of the Chilean law of food labelling and advertising: A nonexperimental prospective study. *PLoS Medicine,**17*(7), e1003220. 10.1371/journal.pmed.1003220 32722710 10.1371/journal.pmed.1003220 PMC7386631

[CR17] Taillie, L. S., Bercholz, M., Popkin, B., Reyes, M., Colchero, M. A., & Corvalán, C. (2021). Changes in food purchases after the Chilean policies on food labelling, marketing, and sales in schools: A before and after study. *Lancet Planet Health,**5*(8), e526–e533. 10.1016/S2542-5196(21)00172-8 34390670 10.1016/S2542-5196(21)00172-8 PMC8364623

[CR18] World Health Organization. (2018). Noncommunicable diseases country profiles 2018. https://www.who.int/nmh/publications/ncd-profiles-2018/en/. Accessed 7 Mar 2020.

